# Simulated aeromedical evacuation exacerbates burn induced lung injury: targeting mitochondrial DNA for reversal

**DOI:** 10.1186/s40779-021-00320-9

**Published:** 2021-05-13

**Authors:** Meng-Jing Xiao, Xiao-Fang Zou, Bin Li, Bao-Long Li, Shi-Jian Wu, Bo Zhang

**Affiliations:** 1 Department of Burn and Plastic Surgery, Air Force Medical Center of Chinese PLA, Beijing, 100142 China; 2 Department of Respiratory and Critical Care Medicine, Air Force Medical Center of Chinese PLA, Beijing, 100142 China

**Keywords:** Aeromedical evacuation, Hypobaric hypoxia, Burn-induced lung injury, Mitochondrial DNA, NLRP3 inflammasome

## Abstract

**Background:**

Aeromedical evacuation of patients with burn trauma is an important transport method in times of peace and war, during which patients are exposed to prolonged periods of hypobaric hypoxia; however, the effects of such exposure on burn injuries, particularly on burn-induced lung injuries, are largely unexplored. This study aimed to determine the effects of hypobaric hypoxia on burn-induced lung injuries and to investigate the underlying mechanism using a rat burn model.

**Methods:**

A total of 40 male Wistar rats were randomly divided into four groups (10 in each group): sham burn (SB) group, burn in normoxia condition (BN) group, burn in hypoxia condition (BH) group, and burn in hypoxia condition with treatment intervention (BHD) group. Rats with 30% total body surface area burns were exposed to hypobaric hypoxia (2000 m altitude simulation) or normoxia conditions for 4 h. Deoxyribonuclease I (DNase I) was administered systemically as a treatment intervention. Systemic inflammatory mediator and mitochondrial deoxyribonucleic acid (mtDNA) levels were determined. A histopathological evaluation was performed and the acute lung injury (ALI) score was determined. Malonaldehyde (MDA) content, myeloperoxidase (MPO) activity, and the nucleotide-binding oligomerization domain-like receptor family pyrin domain-containing 3 (NLRP3) inflammasome level were determined in lung tissues. Data among groups were compared using analysis of variance followed by Tukey’s test post hoc analysis.

**Results:**

Burns resulted in a remarkably higher level of systemic inflammatory cytokines and mtDNA release, which was further heightened by hypobaric hypoxia exposure (*P* < 0.01). Moreover, hypobaric hypoxia exposure gave rise to increased NLRP3 inflammasome expression, MDA content, and MPO activity in the lung (*P* < 0.05 or *P* < 0.01). Burn-induced lung injuries were exacerbated, as shown by the histopathological evaluation and ALI score (*P* < 0.01). Administration of DNase I markedly reduced mtDNA release and systemic inflammatory cytokine production. Furthermore, the NLRP3 inflammasome level in lung tissues was decreased and burn-induced lung injury was ameliorated (*P* < 0.01).

**Conclusions:**

Our results suggested that simulated aeromedical evacuation further increased burn-induced mtDNA release and exacerbated burn-induced inflammation and lung injury. DNase I reduced the release of mtDNA, limited mtDNA-induced systemic inflammation, and ameliorated burn-induced ALI. The intervening mtDNA level is thus a potential target to protect from burn-induced lung injury during aeromedical conditions and provides safer air evacuations for severely burned patients.

## Background

Burns represent a systemic injury, which can induce acute lung injury (ALI), lead to acute respiratory failure, and cause early death in severely burned patients. Even without inhalation injury, major burn patients frequently develop acute respiratory distress syndrome and eventually develop multiple organ failure, often leading to death [1, 2]. The specific mechanism underlying burn-induced lung injury has not been established. Previous studies have indicated that an excessive systemic inflammatory response and the cascading release of inflammatory mediators after burns are among the key factors contributing to burn-induced lung injury [3, 4].

The essence of the inflammatory response after burns and trauma is activation of the innate immune system, which identifies various danger signals through pattern recognition receptors (PRRs) [5–7]. Danger signals released from tissue damage are referred to as damage-associated molecular patterns (DAMPs), such as high mobility group protein 1, uric acid, ATP, hyaluronic acid fragment, and mitochondrial DNA (mtDNA) [8, 9]. Recent studies have shown that mtDNA is one of the key factors mediating innate immunity and the inflammatory response after trauma. Indeed, mtDNA is released into the cytoplasm, extracellular space, or blood following tissue damage or stress and recognized by various types of PRRs, thus activating downstream inflammatory signaling pathways and mediating the systemic inflammatory response [10, 11]. It has been shown that burn injury results in substantial mtDNA release, thereby activating systemic inflammatory pathways and causing ALI. Inhibiting the release of mtDNA is potentially an important target for the treatment of burn-induced lung injuries [12–14].

Air evacuation of severely burned patients is frequent in times of peace and war. The main effect on the body of the air evacuation environment is hypobaric hypoxia. The lung is among the most direct target organs exposed to the hypobaric hypoxia environment; however, how the air environment influences burn-induced lung injuries is unclear. There are an abundance of literature showing that hypoxia is an independent factor contributing to ALI. Moreover, hypoxia can induce an inflammatory response and inflammation can lead to tissue hypoxia, which together cause ALI [15–18]. A hypoxic environment influences the mitochondrial environment. In fact, a lack of oxygen results in mitochondrial dysfunction, produces excessive mitochondrial reactive oxygen species, causes mitochondrial damage, and subsequently results in the release of mtDNA and initiation of a systemic inflammatory reaction [19, 20]. Therefore, the mtDNA-mediated systemic inflammatory response may be a common pathway for burn- and hypoxia-induced lung injuries. Intervention of mtDNA-mediated inflammatory signaling pathway may serve as a therapeutic target to ameliorate burn-induced lung injuries within the air evacuation environment.

Moreover, mtDNA activates diverse PRRs, such as cyclic guanosine monophosphate-adenosine monophosphate synthase (cGAS), the nucleotide-binding oligomerization domain-like receptor family, pyrin domain-containing 3 (NLRP3) inflammasome, and Toll-like receptor 9 (TLR9) [11, 21]. Because the downstream signal molecules are too numerous to intervene, reducing the release of mtDNA from the source is thought to be a direct and effective intervention measure. Therefore, we tested the effect of deoxyribonuclease I (DNase I), a specific endonuclease, on burn-induced mtNDA release and the resulting lung injury in simulated aeromedical evacuation conditions.

## Methods

### Experimental animals and grouping

Male Wistar rats approximately 7 weeks age and weighing (200–250) g, were housed in an animal room with a constant temperature (25 °C) and humidity (50–60)%, and free access to food and water. Animals were housed for at least 7 days prior to the start of experiments. Using NCSS-PASS 11.0.4 software (NCSS, LLC, Kaysville, UT, USA) and assuming a power of 0.9 and an alpha of 0.05, we calculated that 10 animals per group would be required for these experiments. A total of 40 rats were used in this study. Rats were randomly divided into four groups: sham burn (SB) group, burn in normoxia condition (BN) group, burn in hypoxia condition (BH) group, and burn in hypoxia condition with DNase I treatment (BHD) group, each group contained 10 rats. The research protocol was approved by the Committee of Scientific Research of the Air Force Characteristic Medical Center of Chinese PLA (Beijing, China).

### Burn model and treatment intervention

Rats were anesthetized with an intraperitoneal injection of 50 mg/kg of pentobarbital sodium (Sigma-Aldrich, St. Louis, MO, USA). The hairs of the dorsal area were removed and a 30% total body surface area (TBSA) full-thickness burn injury was established by placing the animal in a template exposing 30% TBSA to 100 °C heated water for 10 s. Rats were then resuscitated by intraperitoneal injection of physiologic saline (40 ml/kg) and placed in individual cages. The wounds were treated with topical povidone iodine solution once daily. Rats in the SB group were treated in the same manner, except that the rats were immersed in water at room temperature. Immediately after the burn injury was created, rats in the BHD group received an intravenous injection of DNase I (iv, 8 mg/kg; Sigma-Aldrich, St. Louis, MO, USA). Rats in the BH group received an equal amount of saline solution. To simulate aeromedical evacuation conditions, the rats were placed in a hypobaric hypoxic chamber (simulating 2000 m above sea level) 4 h after the burn injury for 4 h. Immediately after the exposure, animals were euthanized and blood samples were obtained by cardiac puncture. Lung tissues were obtained and fixed by formaldehyde or preserved at − 80 °C for further experiments.

### Detection of plasma mtDNA

The mtDNA was isolated from plasma using a DNeasy Blood and Tissue Kit (Qiagen, Hilden, Germany). All procedures were performed following the manufacturer’s instructions. Briefly, 50 μl of PBS was added to 50 μl of the plasma sample, and the mixture was centrifuged at 16,000×*g* for 15 min at 4 °C. The supernatant was retained. The plasma levels of mtDNA were determined using a SYBR Green Real-time Fluorescent Quantitative PCR Kit (Thermo Fisher Scientific, Waltham, MA, USA). β-actin was used to normalize the data. The rat MT-ND2 gene, a mtDNA gene, was analyzed using the following primers: forward (5′-AAGGAGAGTGGAAGGATGT-3′), and reverse (5′-ATTAGCAGCAGCAGATGG-3′). The sequences of the β-actin primers were forward 5′-GTCAGAAGGACTCCTATGTG-3′ and reverse 5′- ACGCAGCTCATTGTAGAAG-3′. The results were calculated using the 2^–ΔΔCT^ method, where CT represents the cycle threshold value.

### Detection of NLRP3 protein level by Western blotting

Western blotting analysis was performed, as previously described [22]. Membranes were incubated with anti-NLRP3 antibody (Santa Cruz Biotechnology, Inc., Santa Cruz, CA, USA) and the secondary antibody was linked to horseradish peroxidase (1:2000; Invitrogen, Carlsbad, CA, USA). The relative optical density was analyzed.

### Detection of inflammatory cytokines in serum

The concentrations of inflammatory mediators (IL-1β and IL-6) in serum were measured using a specific enzyme-linked immunosorbent assay system (Thermo Fisher Scientific, Waltham, MA, USA) in accordance with the manufacturer’s instructions.

### Assessment of lung injury

Lung tissues were fixed with formaldehyde and stained with hematoxylin and eosin (H&E) before the examination. Five fields were randomly selected in each slice and observed by two independent pathology specialists who were blinded to the experimental groups. The lung injury scoring system was used, as previously described [23]. Alveolar wall thickness, cellular infiltration, and hemorrhage were each scored from 0 (no injury) to 4 (maximal injury). The counts of each score were summed and the results were recorded as the ALI score.

### Detection of malonaldehyde (MDA) content and myeloperoxidase (MPO) activity

Assays were performed according to a commercial kit and the manufacturer’s instructions (Nanjing Jiancheng Bioengineering Institute, Nanjing, China), as previously described [24].

### Statistical analysis

Statistical analysis was performed using GraphPad Prism 6.01 software. Data are presented as the means ± standard deviations (SD). The data among groups were compared using analysis of variance, and the post hoc analysis was performed using Tukey’s test. A *P* < 0.05 was considered statistically significant.

## Results

### The mtDNA level in serum

**Fig. 1:**
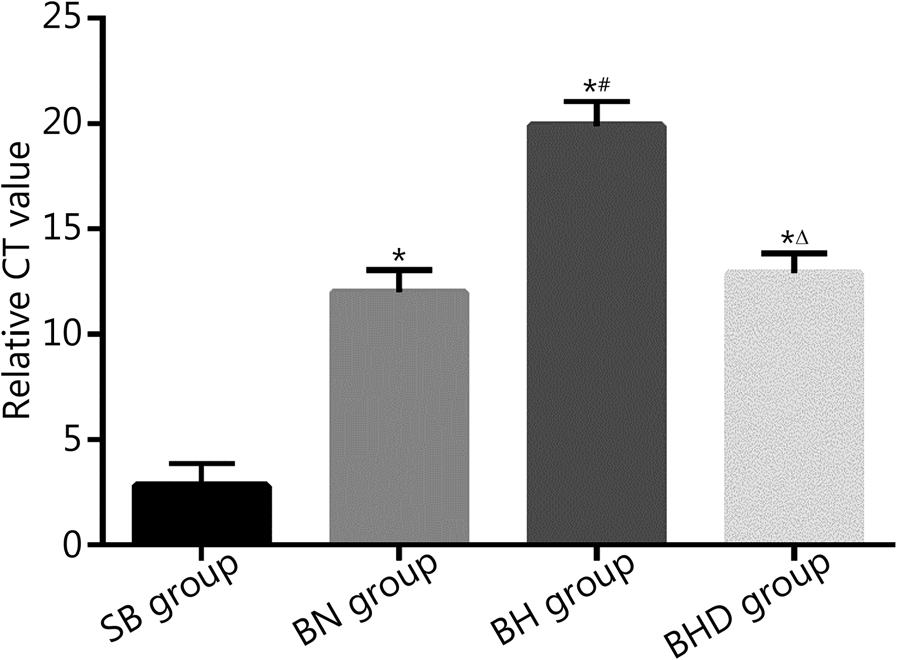
mtDNA levels in serum were detected by quantitative PCR and calculated using the 2^–ΔΔCT^ method, where CT represents cycle threshold value (*n* = 10). mtDNA. Mitochondrial DNA; SB. Sham burn; BN. Burn in normoxia condition; BH. Burn in hypoxia condition; BHD. Burn in hypoxia condition with DNase I treatment. Results are presented as mean ± SD. ^*^P < 0.01 vs. SB group; ^#^*P* < 0.01 vs. BN group; ^△^*P* < 0.01 vs. BH group

The mtDNA level in serum of burned rats was significantly higher than that of sham-burned rats (12.02 ± 1.038 vs. 2.82 ± 1.068, *P* < 0.01). After exposure to hypobaric hypoxia, the mtDNA level (19.92 ± 1.111) was higher than that of the BN group (*P* < 0.01). DNase I treatment significantly reduced the mtDNA level (12.90 ± 0.943) in the serum of burned rats in a hypobaric hypoxia environment (*P* < 0.01, Fig. 1).

### Level of inflammatory cytokines in serum

**Fig. 2:**
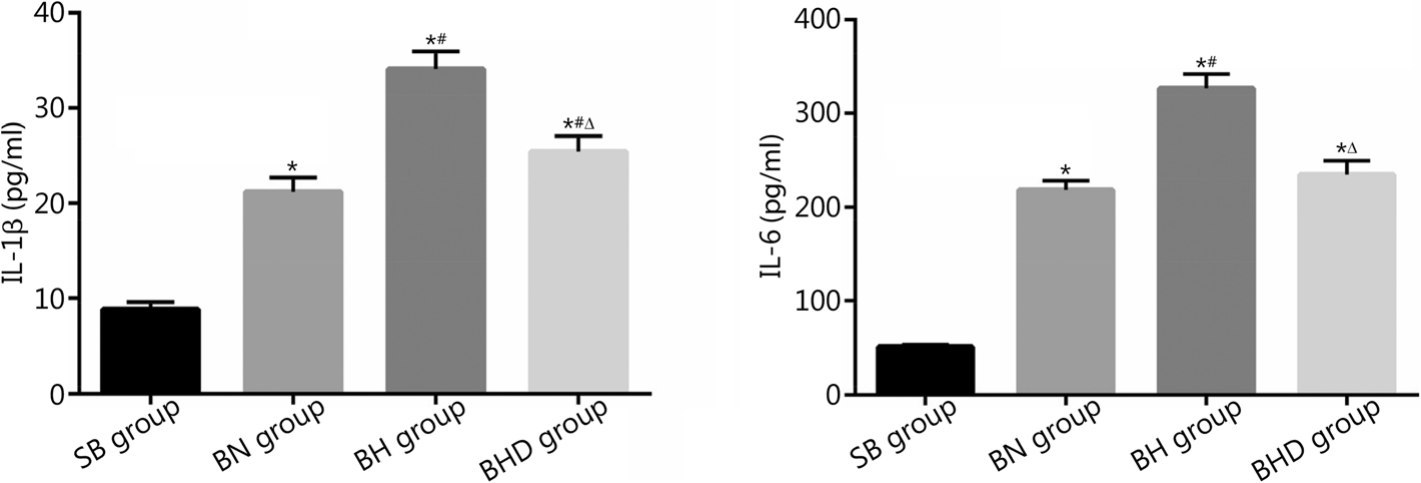
Levels of inflammatory cytokines in serum were detected by ELISA (*n* = 10). SB. Sham burn; BN. Burn in normoxia condition; BH. Burn in hypoxia condition; BHD. Burn in hypoxia condition with DNase I treatment. Results are presented as mean ± SD. ^*^P < 0.01 vs. SB group; ^#^*P* < 0.01 vs. BN group; ^△^*P* < 0.01 vs. BH group

As shown in Fig. 2, the IL-1β levels in serum of SB group, BN group, BH group and BHD group were (8.83 ± 0.796) pg/ml, (21.20 ± 1.498) pg/ml, (34.07 ± 1.855) pg/ml, and (25.46 ± 1.605) pg/ml, respectively; the IL-6 levels were (50.98 ± 2.329) pg/ml, (218.60 ± 9.948) pg/ml, (326.40 ± 15.310) pg/ml, and (234.80 ± 15.090) pg/ml, respectively. Burn injuries resulted in a markedly higher level of IL-1β and IL-6 production in the serum (*P* < 0.01). Inflammatory cytokine production was further elevated when burned rats were exposed to a hypobaric hypoxic environment (*P* < 0.01). DNase I treatment significantly decreased IL-1β and IL-6 production in the serum of burned rats under hypobaric hypoxia conditions (*P* < 0.01).

### MDA content and MPO activity in lung tissues

**Fig. 3:**
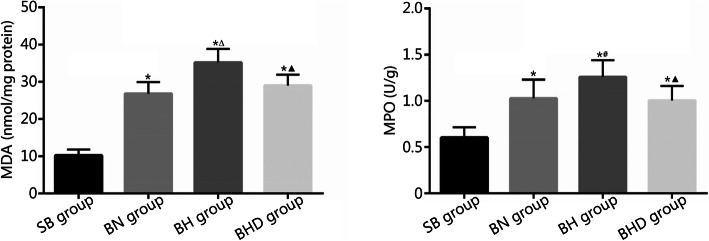
MDA contents and MPO activities in lung tissues were detected according to a commercial kit and the manufacturer’s instructions (*n* = 10). SB. Sham burn; BN. Burn in normoxia condition; BH. Burn in hypoxia condition; BHD. Burn in hypoxia condition with DNase I treatment; MDA. Malonaldehyde; MPO. Myeloperoxidase. Results are presented as mean ± SD. ^*^P < 0.01 vs. SB group; ^#^*P* < 0.05 and ^△^*P* < 0.01 vs. BN group; ^▲^*P* < 0.01 vs. BH group

To evaluate oxidative damage and inflammatory infiltration in lung tissues, we performed experiments to detect the MDA content and MPO activity. As shown in Fig. 3, the MDA contents in lung tissues of SB group, BN group, BH group and BHD group were (10.25 ± 1.621) nmol/mg protein, (26.82 ± 3.123) nmol/mg protein, (35.17 ± 3.636) nmol/mg protein, and (29.04 ± 2.852) nmol/mg protein, respectively; the MPO activities were (0.602 ± 0.114) U/g, (1.028 ± 0.204) U/g, (1.258 ± 0.183) U/g, and (1.005 ± 0.158) U/g, respectively. Burn injuries resulted in a significant elevation of MDA content and MPO activity in lung tissues, which was further heightened by exposure to hypobaric hypoxia (*P* < 0.05 or *P* < 0.01). Moreover, the increases in MDA content and MPO activity were restored by DNase I treatment (*P* < 0.01).

### Histopathological evaluation and assessment of the lung injury

**Fig. 4:**
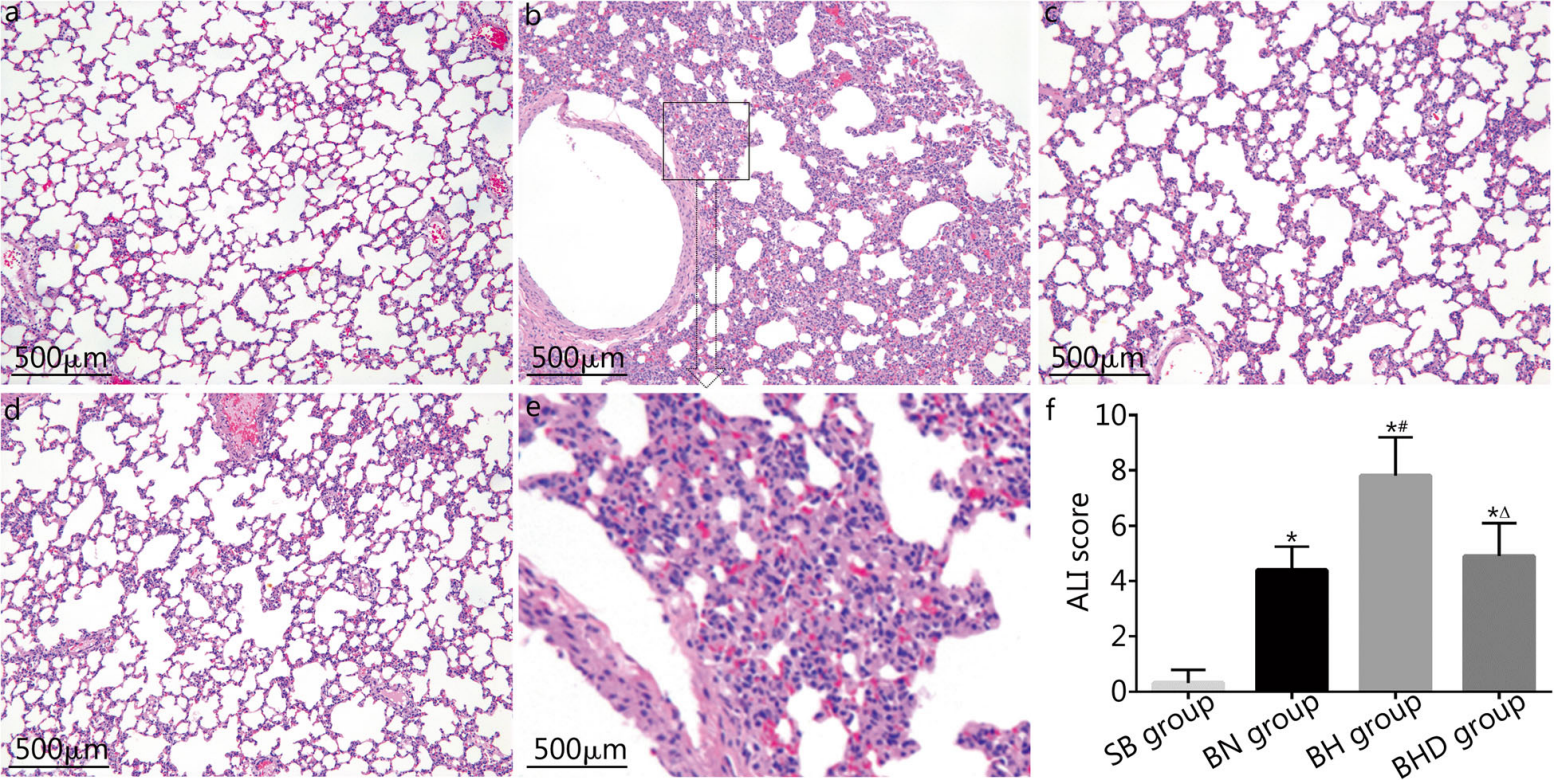
Histopathological examination and assessment of the lung injury (*n* = 10). **a-d** representative images of H&E staining of lung tissues. **a**. SB group; **b**. BH group; **c**. BN group; **d**. BHD group; **e**. Partial enlargement of **b**, showing more severe lung edema, inflammatory infiltration and alveolar hemorrhage. f. ALI scores were calculated and compared among groups. SB. Sham burn; BN. Burn in normoxia condition; BH. Burn in hypoxia condition; BHD. Burn in hypoxia condition with DNase I treatment; ALI. Acute lung injury. Results are presented as mean ± SD. ^*^P < 0.01 vs. SB group; ^#^*P* < 0.01 vs. BN group; ^△^*P* < 0.01 vs. BH group

Lung tissues were stained with H&E and the lung injury was evaluated based on the ALI score. As shown in Fig. 4, rats in the SB group had normal lung tissue structure (Fig. 4a), while rats in the BN group exhibited excessive inflammatory cell infiltration and edematous alveolar walls (Fig. 4c). Furthermore, after rats with burn injuries were exposed to hypoxic conditions, the inflammatory infiltration was increased and the damage to the pulmonary alveoli was more severe (Fig. 4b, e). DNase I treatment attenuated the inflammatory infiltration, reversed edema to the alveolar walls, and ameliorated lung injury (Fig. 4d). The ALI scores in the BN, BH, and BHD group were significantly higher than that of SB group (*P* < 0.01). Moreover, the ALI score was further higher in the BH group than that of the BN group (7.8 ± 1.398 vs. 4.4 ± 0.843), while the ALI score was significantly lower in the BHD group (4.9 ± 1.197) than that of the BH group (*P* < 0.01, Fig. 4f).

### NLRP3 protein level in lung tissues

**Fig. 5:**
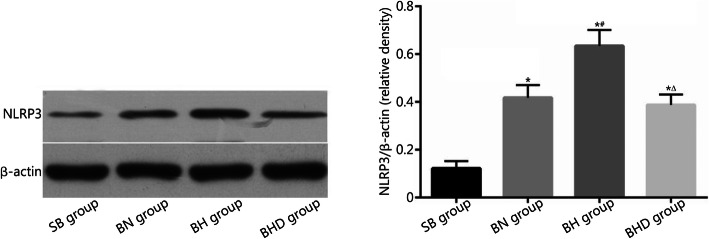
Levels of NLRP3 protein in lung tissues were detected by Western blotting (*n* = 10). SB. Sham burn; BN. Burn in normoxia condition; BH. Burn in hypoxia condition; BHD. Burn in hypoxia condition with DNase I treatment; NLRP3. NLR pyrin domain containing 3. Results are presented as mean ± SD. ^*^P < 0.01 vs. SB group; ^#^*P* < 0.01 vs. BN group; ^△^*P* < 0.01 vs. BH group

The NLRP3 inflammasome is an important downstream signal for mtDNA and recent studies have demonstrated its key role in ALI [25]. Therefore, we designed experiments to determine the level of NLRP3 protein in lung tissues using Western blotting analysis. As shown in Fig. 5, the level of NLRP3 protein in the BN group was significantly higher than that of the SB group (0.418 ± 0.053 vs. 0.122 ± 0.030, *P* < 0.01). Exposure to hypoxia further increased the level of NLRP3 protein in rat lung tissue (0.634 ± 0.066, *P* < 0.01). Moreover, the elevated NLRP3 level was remarkably reduced by DNase I treatment (0.388 ± 0.044, *P* < 0.01).

## Discussion

Aeromedical evacuation is a rapid and effective way to evacuate patients in times of peace and war, however, little is known about the possible effects of aeromedical evacuation on patients with severe burn injuries, especially burn-induced acute lung injuries, which remain a leading cause of early death in severely burned patients. In this study we first observed the effect of a simulated aeromedical evacuation on burn-induced lung injuries, then we explored the possible role of mtDNA-induced inflammation in the pathogenesis of burn-induced lung injuries within a hypobaric hypoxia environment. The results of this study demonstrated that simulated aeromedical evacuation exacerbated burn-induced lung injuries. Moreover, mtDNA mediated systemic inflammation and possibly played an important role in the exacerbation of lung injury. Targeting mtDNA by DNase I ameliorated burn-induced lung injury within a hypobaric hypoxia environment.

There have been a number of studies with a focus on the effect of aeromedical evacuation on different types of injuries, such as traumatic brain injuries, hemorrhagic shock, and blast injuries [26–30]; however, little is known about the effect of aeromedical evacuation on burn injury-related pathophysiology. The most prominent influence of aeromedical evacuation on the human body is hypobaric hypoxia [31]. Previous studies involving traumatic brain or blast injuries have demonstrated that hypobaric hypoxia exacerbates the inflammatory response to these injuries and thus worsens these types of injuries [28, 32]. In the present study, we found the same results as the above-described studies. Burn-induced systemic inflammation and remote lung injuries were also exacerbated by hypobaric hypoxia exposure. These findings supported the notion that hypoxia induces inflammation and causes ALI [15, 18].

To further elucidate why hypobaric hypoxia exposure exacerbated burn-induced systemic inflammation and lung injury, we tested the possible role of mtDNA on the pathogenesis of burn-induced lung injury within a hypobaric hypoxia environment. Indeed, mtDNA was released after tissue injury and played a crucial role in the development of inflammation after injury. Studies have shown that mtDNA induces inflammatory responses and causes lung injury in thermal injury murine models [13, 14]. Furthermore, the mitochondria are among the most sensitive target organs when exposed to hypoxia. Hypoxia leads to mitochondrial dysfunction and induces the release of a variety of DAMPs, such as nuclear high mobility group box 1 and mtDNA [33, 34]. Therefore, it follows that hypobaric hypoxia increases burn injury-induced mtDNA release, which was confirmed by the results of the present study.

mtDNA induces inflammatory response through various receptors and related downstream signaling pathways, including cGAS, NLRP3, and TLR9. We hypothesized that administering external DNase I, a nuclease responsible for degrading extracellular DNA, can reduce mtDNA release from the source, and thus abrogate mtDNA-induced inflammation and ameliorate remote lung injury. The results of this study showed that a single dose of DNase I (8 mg/kg) significantly reduced the mtDNA level in serum, suppressed the NLRP3 inflammasome level in lung tissues, and ameliorated lung injury in a rat burn model. These results are of significance because recombinant human DNase I has been used in the clinical setting for patients with pulmonary diseases, such as cystic fibrosis and pneumonia, and has shown excellent safety and efficacy [35, 36]; however, the mechanisms underlying these protective effects are likely different from the actions reported herein. We speculate that the above mentioned effects antagonize mtDNA signaling, at least in part; this speculation warrants further investigation. Moreover, if the results of this study can be extrapolated to the clinical setting, the efficacy and safety of DNase I should be validated in larger animals and humans. At that time, it is recommended that DNase I be administrated in a way close to the practical clinical use, such as intratracheal administration.

## Conclusions

The results in this study suggested that simulated aeromedical evacuation further increased the burn-induced mtDNA release and exacerbated burn-induced inflammation and lung injury. DNase I reduced the release of mtDNA, limited mtDNA-induced systemic inflammation, and ameliorated burn-induced ALI. Inhibition of mtDNA could be a potential therapeutic target to protect burn-induced lung injuries during aeromedical conditions and provides safer air evacuations for severely burned patients.

## Data Availability

The datasets used and/or analysed during the current study are available from the corresponding author on reasonable request.

## Abbreviations

ALI: Acute lung injury
cGAS: Cyclic guanosine monophosphate-adenosine monophosphate synthase
DAMPs: Damage associated molecular patterns
DNase I: Deoxyribonuclease I
MDA: Malonaldehyde
MPO: Myeloperoxidase
mtDNA: Mitochondrial DNA
NLRP3: NLR pyrin domain containing 3
PRRs: Pattern recognition receptors
TBSA: Total body surface area
TLR9: Toll like receptor 9
